# Spatial and spatio-temporal distribution of women living with HIV mortality in Porto Alegre, Brazil, from 2007 to 2017

**DOI:** 10.11606/s1518-8787.2021055003384

**Published:** 2021-11-23

**Authors:** Maiton Bernardelli, Tonantzin Ribeiro Gonçalves, Marcos Pascoal Pattussi, Nêmora Tregnago Barcellos, Lisiane Acosta

**Affiliations:** I Universidade do Vale do Rio do Sinos Programa de Pós-Graduação em Saúde Coletiva São Leopoldo RS Brasil Universidade do Vale do Rio do Sinos. Programa de Pós-Graduação em Saúde Coletiva. São Leopoldo, RS, Brasil; II Centro Universitário da Serra Gaúcha FSG Caxias do Sul RS Brasil Centro Universitário da Serra Gaúcha FSG. Caxias do Sul, RS, Brasil

**Keywords:** Women, HIV Infections, mortality, Risk Factors, Socioeconomic Factors, Health Status Disparities, Ecological Studies

## Abstract

**OBJETIVE:**

To present some factors related to the mortality rates of WLHIV in the city of Porto Alegre-RS.

**METHODS:**

This is a spatial and spatio-temporal analysis of ecological data about all women monitored by the health care services for the vertical transmission (VT) of HIV, between 2007 and 2017, residing in the city that died during the period. The units of analysis were the 17 sanitary districts of the city. The dependent variable was the mortality rate. The independent territorial variables were the indicators of vulnerability to poverty, women householder proportion, lack of infrastructure, HDI, and GINI index. Still, the individual data collected were: age, race/color, level of education, and period since the HIV diagnosis. The analyses used SPSS 20.0, and QGIS 218.15.

**RESULTS:**

Regions with higher vulnerability to poverty and precarious local infrastructure registered higher WLHIV mortality rates, especially black/”pardo” women in fertile age with low education. The regions with most women householders presented a risk of mortality seven times higher. The population with vulnerability to poverty presented the same result.

**CONCLUSIONS:**

Regions with critical indicators of vulnerability presented higher mortality rates of WLHIV, which demonstrates social inequalities’ impact for these women.

## INTRODUCTION

Data from the Joint Unites Nations Programme on HIV and AIDS (UNAIDS) indicate that around 38 million people were infected by human immunodeficiency virus (HIV) all over the world in 2019. African countries registered the higher number of people living with HIV. New infections affected especially teenagers and adult women. Among Latin American and Caribbean countries, Brazil has most cases, with approximately 920 thousand people living with HIV (PLHIV) in 2019^1^.

Between advances and setbacks, Brazil still stands out internationally regarding its response to the HIV epidemic response and the acquired immunodeficiency syndrome (AIDS) caused by the virus. It occurs mainly by the universal access to antiretroviral therapy (ART) through the Brazilian Unified Health System (SUS) since 1996^2^. During these more than thirty years fighting the epidemic, HIV detection rates oscillated significantly^3^, presenting regional variations according to the country's states^4^. South and Southeast regions present the higher concentration of women living with HIV (WLHIV)^5^. The South region particularly has the higher percentage of women with AIDS, and the ratio between genders is 18 men for each 10 women^6^.

Porto Alegre, capital of the state of Rio Grande do Sul (RS), reached the third highest AIDS detection rate (53.7/100 thousand inhab.) among Brazilian capitals and the Federal District, as well as the higher rate of mortality by AIDS (22.5/100 thousand inhab.)^6^. From the total cases of AIDS registered in the state of Rio Grande do Sul between 1980 and 2015, 40.6% were women, indicating an increase of women infected in the period^7^. Moreover, the mortality rate by AIDS in Porto Alegre is 3.5 times higher than in the state (7.8/100.000 inhab.), which is higher than the national average (4.4/100.000 inhab.)^6^ and directly impacts the female population between 30 and 39 years old^7^.

Porto Alegre has 1,409,351 inhabitants with a predominant female population in the age range between 20 and 29 years old^8^. By the high rates of HIV detection, the city faces several challenges to confront the epidemic, especially regarding health inequalities and late diagnosis^9^. The absence of accurate morbidity and mortality data is a main challenge to monitor and evaluate local responses, impeding evidence-based strategies to prevent mortality in WLHIV^10^.

The main structural determinants impacting HIV/AIDS in Brazil are gender inequalities, race, and impoverishment of WLHIV^11^. Therefore, mortality analysis models for PLHIV are important to consider the influence of health inequalities and their determinants on this population mortality. This article aims to present a spatial and spatio-temporal analysis of the mortality of WLHIV in Porto Alegre between 2007 and 2017, investigating contextual aspects associated with the city's different sanitary districts.

## METHODS

This is a multilevel ecological study comprising the 17 Sanitary Districts (SD) of Porto Alegre: Ilhas, Humaitá/Navegantes, Centro, Noroeste, Norte, Eixo Baltazar, Leste, Nordeste, Glória, Cruzeiro, Cristal, Sul, Centro-Sul, Partenon, Lomba do Pinheiro, Restinga, and Extremo-Sul. According to the 2010 census, Porto Alegre had a population of 1,409,351 inhabitants, of which 53.6% were women.

The population studied comprised all WLHIV monitored by the health care services for the vertical transmission (VT) of HIV in the city that died between 2007 and 2017. The database of the VT monitoring service provided the individual data, which were obtained from the epidemiological surveillance of the Porto Alegre Health Secretariat. The database stores data from the *Sistema de Informação de Agravos de Notificação* (SINAN – Information System of Aggravation Notification) about pregnant women with HIV. Mortality rate data were obtained from the *Sistema de Informação sobre Mortalidade* (SIM – Information System about Mortality), provided by Porto Alegre city hall. Data linkage was used to integrate data, using the name of the pregnant woman registered in SINAN and SIM as the key-variable. Birthdate and mother's name were used to verify the adequacy of pairs.

The codes of the 10th revision of the International Statistical Classification of Diseases and Related Health Problems (ICD-10) described in the field “primary cause” of the death certificate (DC) were used to analyze the mortality causes. To calculate the specific mortality rate for WLHIV, we used the absolute frequency of these causes of death in each DC divided by the total of women in fertile age (10 to 49 years)^8^, multiplied by 100,000.

Two analyses were conducted, the first about potential contextual variables related with the mortality rates in different sanitary districts, and the second about spatio-temporal distribution of the cases in the city. The contextual variables included: a) Gini index; b) proportion of vulnerability to poverty equivalent to a family with an income of 1/2 minimum wage (R$255,00) per resident; c) percentage of people in households without water supply and inadequate sewage system; d) percentage of the population living in urban households without waste collection; e) percentage of the population living in households without electrical energy; f) proportion of women householders living in social vulnerability; g) human development indices (HDI–2010) measured by the geometrical mean of three sub-indices with equal weights (longevity and life expectancy at birth; per capita income; education/frequency of children and teenagers at school - 2/3 – and adult population's level of education - 1/3). The indicators were obtained from the *Atlas de Desenvolvimento Humano do Brasil para 2010* (http://atlasbrasil.org.br). After the analysis of correlation, the SDs were grouped in four strata according to each explanatory variable with high correlation with the mortality rates. The distribution of frequencies and correlations was conducted with the software SPSS 20.0.

For the spatial distribution, we used the mortality points of occurrence obtained in the registers of SIM present in the DC, using the fields “name” and “address”. Afterward, we located the addresses using Google Earth Pro and marked them in the units of analysis to which they pertained. A project was created in the *Sistemas de Informações Geográficas* (SIG) QGIS 218.15, where the reference system was configurated for the plain coordinates: Universal Transverse Mercator (UTM), and Datum SIRGAS2000. The analysis of density in the points was verified using Kernel method, with an interpolator using a radius of 100 meters.

The Research Ethical Committee of the Universidade do Vale dos Sinos (UNISINOS – opinion n. 3.233.242) and Porto Alegre Health Secretariat (SMS/POA – opinion n. 3.281.948) approved the research.

## RESULTS

Among all WLHIV in the city, 3,164 women were monitored by the monitoring system of HIV viral transmission between 2007 and 2017, and 121 deaths were registered during this period. The analysis excluded one case by the lack of data regarding address and reference to the unit of analysis. The highest percentages of these deaths occurred with women between 30 and 39 years old (43%), black or “pardo” (mixed ethnicity) (55,4%), between one and seven years of education (57%), and period since the HIV diagnosis equal or lower than five years before the death (62%). All cases investigated had at least four pregnancies during their lifetime.

Among the causes of death, diseases related to HIV were the primary cause, representing 77.7% (N = 94) of deaths. The other 33.3% (N = 27) of cases had varied causes, such as violence (gun, knife, or physical aggression), maternal death, neoplasm, cardiovascular diseases caused by disorders or abuse of psychoactive substances, liver cirrhosis, and septic arthritis.

**Figure 1:**
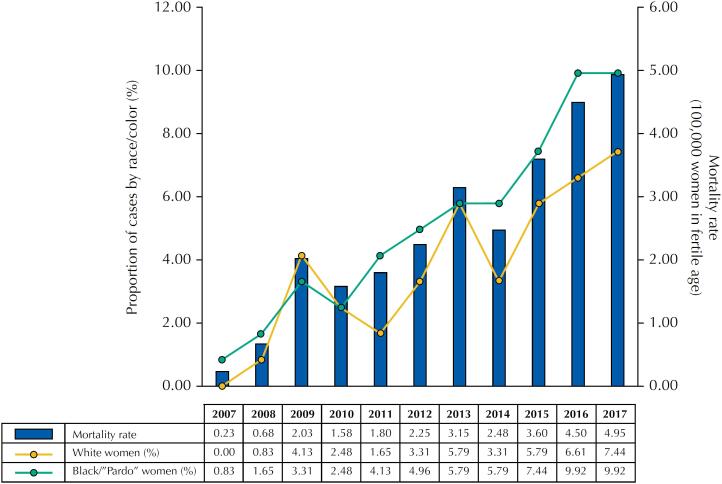
Mortality rate and proportion of cases by race/color in WLHIV notified by SINAN in Porto Alegre/RS between 2007 and 2017.

A progressive increase of deaths occurred during the years. The mortality rate was 0.23/100,000 in 2007 and 4.95/100,000 women in fertile age in 2017. The higher proportion of cases occurred with black or “pardo” WLHIV, except in 2009 where the higher proportion occurred among white women (Figure 1).

Through the whole period, the higher mortality rate occurred in the SD Ilhas, where 117.83 deaths per 100,000 women in fertile age occurred. It was followed by the SD Nordeste, Humaitá-Navegantes, Lomba do Pinheiro, and Cruzeiro. The lower rate occurred in the region Centro Sul with 6.32 deaths per 100,000 women in fertile age (Table 1).

**Table 1:** Mortality rate distribution of WLHIV notified in SINAN for Porto Alegre/RS and its Sanitary Districts between 2007 and 2017^a^.

Units of Analysis	Women in fertile age (10–49)^b^	2007	2008	2009	2010	2011	2012	2013	2014	2015	2016	2017	2007 to 2017
PORTO ALEGRE	444,236	0.23	0.68	2.03	1.58	1.80	2.25	3.15	2.48	3.60	4.50	4.95	27.24
Ilhas	2,546						78.55			39.27			117.83
Nordeste	11,840				8.44		8.44	25.33		8.44	8.44	16.89	76.02
Humaitá/Navegantes	14,690		6.80	6.80	6.80	13.61		6.80		13.61	13.61	6.80	74.89
Lomba do Pinheiro	18,967			5.27	5.27	10.54		5.27	15.81		15.81	15.82	73.81
Cruzeiro	18,858				5.30	5.30	10.60	10.60	5.30	10.60	5.30	5.30	58.33
Leste	35,249			2.84	2.84	2.84	2.84	5.67		8.51	5.67	5.67	36.89
Partenon	35,701		2.80		2.80		5.60	8.40			8.40	5.60	33.61
Eixo Baltazar	30,610					3.27	3.27	3.26	6.53	3.27		9.80	29.40
Glória	18,187		5.50	5.50					5.50	5.50			21.99
Norte	32,648								3.06		12.25	6.12	21.44
Extremo Sul	10,583										9.45	9.45	18.89
Sul	27,781			3.60	3.60				3.60	7.20			17.99
Noroeste	40,124					2.50					4.99	4.99	12.47
Centro	85,050	1.17		3.53				1.17	2.35	2.35		1.17	11.76
Restinga	19,484						5.13				5.13		10.26
Cristal	10,276							9.73					9.73
Centro Sul	31,643			3.16						3.16			6.32

a For each 100,000 women in fertile age.

b IBGE – 2010.

Spearman's rank correlation coefficient indicated moderate correlation between the mortality rate of WLHIV and the proportion of women householder by residence in the SDs and the proportion of people vulnerable to poverty (Table 2).

**Table 2:** Spearman's rank correlation coefficients (p) between WLHIV mortality rates and contextual variables of Porto Alegre's Sanitary Districts.

Indicator/Rate^a^	Proportional Mortality Rate 2007 to 2017 (p)	CI95%^b^	p
GINI index	0.059	− 0.462–0.586	0.823
% population vulnerable to poverty	0.558	− 0.031–0.872	0.020
% households without water and sewage	0.488	0.009–0.842	0.047
% households without waste collection	0.543	0.036–0.851	0.024
% households without electrical energy	0.281	− 0.253–0.720	0.275
% women householders in vulnerability	0.698	0.215–0.925	0.002
HDI-M	− 0.583	− 8.65–0.035	0.014
HDI-M – Education	− 0.562	− 0.878–0.015	0.019
HDI-M – Longevity	− 0.465	− 0.833–0.120	0.060
HDI-M – Income	− 0.502	− 0.808–0.045	0.040

HDI-M: Municipal Human Development Index.

a Atlas do Desenvolvimento Humano, 2010.

b Bootstrap by 1,000 observations.

**Figure 2:**
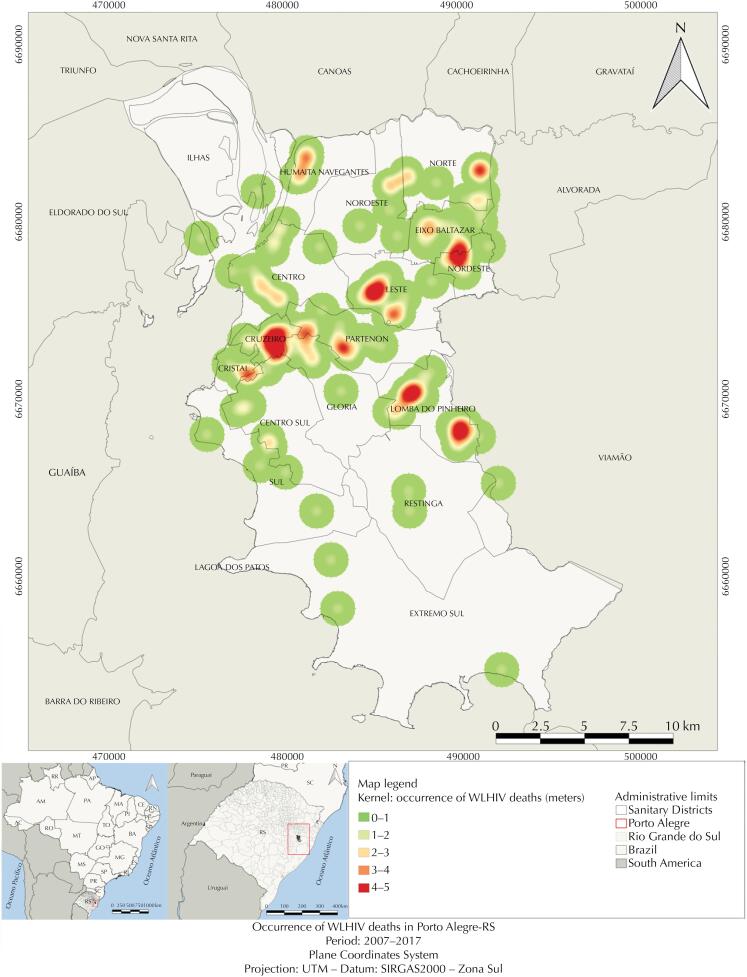
Kernel map of WLHIV mortality cases in Porto Alegre (2007–2017).

The Kernel map (Figure 2) identified hot spots in the SDs Cruzeiro, Lomba do Pinheiro, Leste, and Nordeste. The areas with lower density were SD Extremo Sul, Centro Sul, and Ilhas.

**Figure 3:**
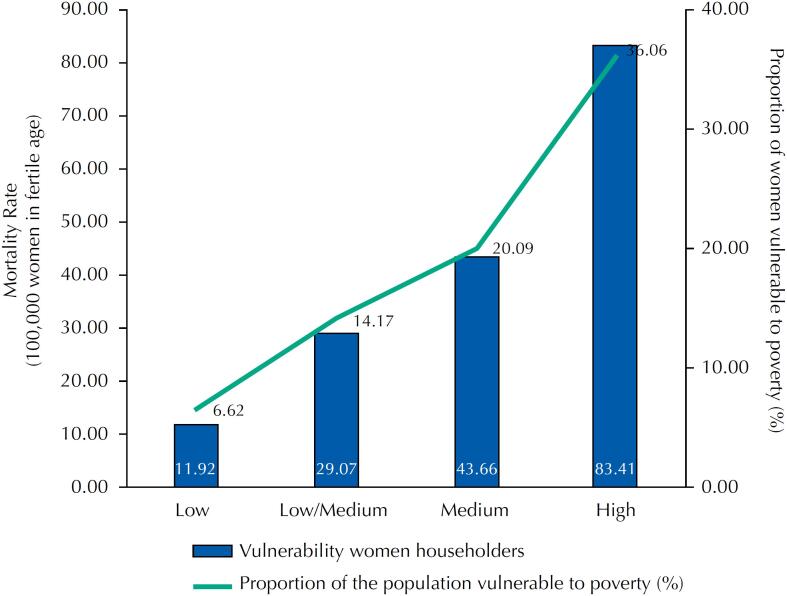
Distribution of WLHIV mortality rates according to the percentage of Porto Alegre's women householders in vulnerability (2007–2017).

The aggregate of mortality rates concerning the percentage of women householders in vulnerability demonstrated a gradient with higher rates in districts with higher vulnerability. The rates were 11.92/100,000 for women with lower vulnerability and 83.4/100,000 for women with higher vulnerability (Figure 3). Such results indicate that the SDs included in stratum 4 (Nordeste and Ilhas) had a mortality rate seven times higher than stratum 1 (Centro, Nordeste, Centro Sul, and Sul). The SDs Nordeste and Ilhas have, respectively, 37.65% and 34.46% of their population vulnerable to poverty, evidencing the impoverishment of such women.

## DISCUSSION

Although improvements occurred since the implementation of universal access to antiretroviral therapy (ART) in Brazil since 1996, the situation of WLHIV mortality in Porto Alegre reveals significant social inequality between its SDs. Contextual vulnerabilities, such as poverty and housing conditions, and the high number of women householders with young children living in the SDs with high WLHIV mortality rate are significant examples of social inequality. Another study also identified the impact of vulnerability in deaths related to HIV/AIDS, which occurred mostly with younger patients with low socioeconomic conditions^12^.

The precocity of the deaths is relevant as the higher incidence of deaths occurred with women between 30 and 39 years old. National data about the South region^6^ and the state of Rio Grande do Sul^7^ corroborate this finding. A study evaluating the magnitude and trend of AIDS mortality between 2000 and 2011 (also in Rio Grande do Sul and Porto Alegre) found an increase of women in fertile age's deaths of 4.1% (CI95% 3.0–5.3) in the state and 2.7% (CI95% 1.8–3.5) in Porto Alegre; the high rates possibly reflect the addition of social inequalities to the inequality of access to health care services^13^.

Porto Alegre has challenges in Primary Health Care (PHC) management, which possibly reflect in the high mortality rate. Peripheral areas with high violence rates have less health professionals than central areas, which leads to vulnerable areas with a lack of health care^14^. Between 2006 and 2010, Brazilian cities with intense AIDS epidemic presented risk contexts related to gender differences and high rates of alcohol and drug abuse^9^. Such context challenges HIV/AIDS-related policies, especially for WLHIV.

Regarding the particular characteristics studied here, the Kernel map presented hotspots in more vulnerable regions with higher percentage of low income population^8^, especially the SD Cruzeiro. This region has a high concentration of substandard housing, indicating inequality in the distribution of the urban structure and the precarity of its inhabitants’ living conditions^15^. According to the 2010 Census^8^, the regions of SDs Lomba do Pinheiro and Nordeste have HDI below the national average, including the aspects longevity, education, and income.

Population indices show that the higher concentration of the black population in Porto Alegre (around 27%^8^) lives in regions lacking basic resources and sanitation^17^. Such conditions negatively impact the health of women living in the regions Nordeste, Leste, Cruzeiro, and Lomba do Pinheiro. This fact is relevant considering that the mortality is higher among black and “pardo” WLHIV, which shows this population vulnerability and the effects of structural racism in Brazil^13-16^, reflected in the unequal access to health care^17^.

Aspects such as race/color may influence the report of the death cause in DCs, leading to distortions^18,19^, which consequently weaken actions for vulnerable populations due to processes of institutional racism in the society^16^. In USA, for instance, the proportional mortality rate of the black population in 2010 was ten times higher than the White population living with HIV^18^. A study in Brazil found similar results, where non-whites had more registers of death by HIV/AIDS than white individuals^20^. At the same time, studies found that death records of white persons tend to be unrelated to HIV/AIDS, closer to the general population profile^19^.

HIV/AIDS-related diseases were the main cause of death in the cases reported in this study. This fact corroborates a Canadian study that identified HIV/AIDS as the main cause of death for PLHIV, and the relative risk of death remains higher than in non-infected individuals^21^. Besides the advancements in diagnosis and treatment, 73.3% (639/868) of PLHIV deaths in a health unit of Rio de Janeiro between 1986 and 2009 were AIDS-related^22^. Nevertheless, this percentage reduced from 86.7% to 61.7% in the same period. In the state of Pernambuco, 74% (232/315) of the deaths studied were HIV-related^23^.

In our study, the second higher cause of death was gun or knife crimes, followed closely by maternal deaths. Such violent deaths stress the vulnerability to violence in the daily life of WLHIV in Brazil^24,25^.

The regions Ilhas and Nordeste have high rates of low income and householder women, and the mortality of WLHIV follow this trend. In these regions, according to the *Atlas do Desenvolvimento Humano* (2010), 1/4 of women are the only providers of the household, have incomplete basic education, and at least one child until 15 years old living in the household.

This result reflects, on the one hand, the increasing importance of women in family subsistence, and, on the other hand, significant inequalities because many women are the sole providers for their children basic needs^26^. Many women in such conditions are exposed to other risk situations, such as drug trade and other violence contexts^26^. Challenges related to drug abuse^27^ may strengthen structural barriers to health care access, especially those regarding early HIV diagnosis and treatment^28^, which increases WLHIV mortality risk^29^.

Thus, the challenges caused by such vulnerabilities should be taken into account when they are followed by short-range public policies and health services to mitigate these inequalities^10,30^. In this sense, a study^10^ analyzing characteristics and therapeutic itineraries in AIDS-related deaths by the *Comitê Municipal de Mortalidade por Aids de Porto Alegre* (Porto Alegre's AIDS-related mortality committee) in 2015 concluded that failures in health care organization and lack of access were determinant in the deaths studied. Therefore, there is a process of accumulation, maintenance, and increase of disadvantages during WLHIV's life trajectories. Actions to reduce mortality require efforts considering the interrelation between psychosocial, cultural, and sociopolitical factors^11^. National, local, and regional characteristics shall also be interrelated to minimize vulnerabilities, and qualify aggravation prevention and health care for WLHIV.

Nevertheless, some limitations shall be considered. The main limitation of ecological studies is that association among exposure and outcome is not possible. SDs as units of analysis have heterogeneous characteristics. Nevertheless, they are defined by SUS and adopted by Porto Alegre's public health management. Thus, its adoption favors decision-making and actions planning for priority regions. Also, data dispersion and heterogeneity impeded the analysis of spatial autocorrelation. However, the Kernel method identified SDs with hot spots for mortality cases. Underreporting and notification errors are common in secondary bases and can affect data quality. Still, the low number of absent data in death reports denote a qualification of data sources used by the city's epidemiological surveillance.

Additionally, other HIV-related causes of death, such as tuberculosis, were not analyzed because eventual errors in reporting could hamper the matching of data with other databases provided by SINAN. Moreover, the results reflect specific contextual questions related to the variables used, which cannot be extrapolated to an individual level. Assuming social, access, and services inequalities in Porto Alegre/RS as modifiable aspects, this study performed a spatial and a spatio-temporal analysis of WLHIV mortality monitored by the health care service for HIV vertical transmission in the city. Thus, we analyzed contextual and epidemiological aspects of the SDs. The regions with higher social vulnerability presented hot spots of WLHIV deaths, showing the impact of social inequalities in the mortality rates.

Thus, it is crucial to expand public policies efforts in health care and HIV-prevention, especially in policies beyond reproductive matters^24^, including integral and longitudinal health care of women in general, and WLHIV in particular. We hope that this and other studies will help increase health care actions for WLHIV, proposing strategies beyond individual interventions and stimulating public policies that guarantee a dignified life with access to education, work, housing, income, and health services.
