# Supplementary material for: Spatial and spatio-temporal distribution of women living with HIV mortality in Porto Alegre, Brazil, from 2007 to 2017
Source: Rev Saude Publica. 2021 Nov 23;55:84. doi: 10.11606/s1518-8787.2021055003384 (PMC8621530; doi:10.11606/s1518-8787.2021055003384)
Supplement: Translation

# Translation: Distribuição espacial e espaço temporal de óbitos de mulheres vivendo com HIV em Porto Alegre de 2007 a 2017

**Authors:** Maiton Bernardelli, Tonantzin Ribeiro Gonçalves, Marcos Pascoal Pattussi, Nêmora Tregnago Barcellos, Lisiane Acosta

**Affiliations:** I São Leopoldo RS Brasil Universidade do Vale do Rio do Sinos. Programa de Pós-Graduação em Saúde Coletiva. São Leopoldo, RS, Brasil; II Caxias do Sul RS Brasil Centro Universitário da Serra Gaúcha FSG. Caxias do Sul, RS, Brasil

**Keywords:** Mulheres, Infecções por HIV, mortalidade, Fatores de Risco, Fatores Socioeconômicos, Disparidades nos Níveis de Saúde, Estudos Ecológicos

## Abstract

**OBJETIVO:**

Apresentar fatores associados à taxa de óbitos de mulheres vivendo com HIV (MVHIV) na cidade de Porto Alegre-RS.

**MÉTODOS:**

Estudo ecológico de análise espacial e espaço temporal incluindo todas as mulheres acompanhadas nos serviços de atenção à transmissão vertical (TV) do HIV, entre 2007 e 2017, residentes no município e que faleceram nesse período. As unidades de análise foram os 17 distritos sanitários do município. A variável dependente foi a taxa de óbito e as independentes territoriais foram os indicadores de vulnerabilidade à pobreza, proporção de mulheres chefe de família, escassez de infraestrutura, IDH e o índice de GINI, já as advindas da informação individual foram: idade, raça/cor, escolaridade, tempo de diagnóstico HIV. As análises utilizaram o SPSS 20.0 e o QGIS 218.15.

**RESULTADOS:**

Taxas mais elevadas de óbito de MVHIV foram registradas nas regiões com maior vulnerabilidade à pobreza e precariedade na infraestrutura local, associadas ao desfecho em mulheres em idade fértil, pretas/pardas e de baixa escolaridade. Nas regiões com altas taxas de mulheres que chefiam a família, a prevalência de óbito foi sete vezes maior, mesmo resultado obtido com a proporção populacional de vulnerabilidade à pobreza.

**CONCLUSÕES:**

As regiões que apresentam indicadores de vulnerabilidade críticos resultaram em taxas mais elevadas de óbito em MVHIV, revelando o impacto das desigualdades sociais em saúde para a morte dessas mulheres.

## INTRODUÇÃO

Dados das Nações Unidas sobre HIV/aids (UNAIDS) indicam que havia cerca de 38 milhões de pessoas infectadas pelo vírus da imunodeficiência humana (HIV) no mundo em 2019. Países da África registram o maior número de pessoas vivendo com o vírus, sendo meninas e mulheres jovens as mais impactadas pelas novas infecções. Já entre os países da América Latina e Caribe, o Brasil lidera o ranking com uma estimativa de, aproximadamente, 920 mil pessoas vivendo com HIV (PVHIV) no país em 2019^1^.

Entre avanços e retrocessos, o Brasil permanece sendo destaque mundial no enfrentamento à epidemia do HIV e da síndrome de imunodeficiência adquirida (aids) causada pelo vírus, especialmente pela implantação do acesso universal da terapia antirretroviral (TARV) através do Sistema Único de Saúde (SUS), desde 1996^2^. Nos mais de trinta anos de enfrentamento da epidemia, as taxas de detecção do HIV e da aids sofreram oscilações importantes^3^, com diferenças regionais entre os estados da federação^4^. As regiões sul e sudeste vem apresentando, ao longo dos anos, as maiores concentrações dos casos de mulheres vivendo com HIV (MVHIV)^5^.A região sul, em particular, apresenta maior percentual de mulheres com aids, onde a razão entre os sexos está em 18 homens para cada 10 mulheres^6^.

Porto Alegre, capital do Rio Grande do Sul (RS), atingiu a terceira maior taxa de detecção de casos de aids (53,7/100 mil hab.), entre as capitais brasileiras e Distrito Federal, além das maiores taxas de mortalidade por aids (22,5 por 100 mil hab.)^6^. Do total de casos de aids registrados no Rio Grande do Sul entre os anos 1980 e 2015, 40,6% foram de mulheres, apontando nítido aumento de casos em mulheres ao longo dos anos^7^. Ademais, a taxa de mortalidade por aids em Porto Alegre é 3,5 vezes superior à observada no estado (7,8/100.000 hab.), superando a taxa nacional (4,4/100.000 hab.)^6^, impactando diretamente a população feminina com idade entre 30 e 39 anos^7^.

Com uma população de 1.409.351, o município de Porto Alegre se caracteriza pelo predomínio da população feminina na faixa etária de 20 a 29 anos^8^. Pelos altos índices de detecção do HIV, o município apresenta inúmeros desafios no enfrentamento à epidemia, especialmente relacionados às iniquidades em saúde e ao diagnóstico tardio^9^. A ausência de informações acuradas sobre morbidade e mortalidade se destacam como barreira ao monitoramento e avaliação das respostas locais, inviabilizando estratégias baseadas em evidências e focadas na prevenção dos óbitos de PVHIV^10^.

Como determinantes estruturais, as desigualdades de gênero, raça e pauperização das MVHIV impactam no cenário do HIV/aids no país^11^ e, portanto, é relevante destacar modelos de análise da mortalidade em PVHIV que considerem a influência de questões epidemiológicas à luz das desigualdades em saúde e seus determinantes sobre os óbitos dessa população. O artigo objetiva apresentar uma análise espacial e espaço temporal de mortes de MVHIV na cidade de Porto Alegre, entre 2007 e 2017, e investigar aspectos contextuais associados em diferentes distritos sanitários do município.

## MÉTODOS

Trata-se de estudo ecológico multinível que abrangeu os 17 Distritos Sanitários (DS) do município de Porto Alegre: Ilhas, Humaitá/Navegantes, Centro, Noroeste, Norte, Eixo Baltazar, Leste, Nordeste, Glória, Cruzeiro, Cristal, Sul, Centro-Sul, Partenon, Lomba do Pinheiro, Restinga e Extremo-Sul. Conforme o censo de 2010, o município de Porto Alegre possuía uma população de 1.409.351 habitantes, sendo 53,6% mulheres.

A população do estudo foi composta por todas as MVHIV acompanhadas pelos serviços de atenção à transmissão vertical (TV) do HIV no município e que faleceram entre 2007 e 2017. Dados individuais foram obtidos a partir do banco de informações do serviço de monitoramento da TV, junto à vigilância epidemiológica da Secretaria de Saúde de Porto Alegre, que recebe os dados do Sistema de Informação de Agravos de Notificação (SINAN) de gestantes vivendo com HIV. Os dados dos óbitos foram obtidos junto ao Sistema de Informação sobre Mortalidade (SIM), fornecido pela Prefeitura Municipal de Porto Alegre. A vinculação dos dados foi realizada por meio de *linkage*, sendo escolhida como variável-chave o nome da gestante constante nos registros do SINAN e SIM. Para constatar adequação dos pares verificaram-se as datas de nascimento e o nome da mãe da gestante.

Para análise das causas de óbito, foram considerados os códigos da 10ª Revisão da Classificação Estatística Internacional de Doenças e Problemas Relacionados à Saúde (CID-10) descritos no campo “causa básica” constantes na Declaração de Óbito (DO). Para cálculo da taxa de mortalidade específica para MVHIV considerou-se a frequência absoluta desses óbitos em cada DS, dividida pelo total de mulheres em idade fértil (10 a 49 anos)^8^, multiplicada por 100.000.

Foram realizadas duas análises, uma sobre potenciais variáveis contextuais associadas às taxas de mortalidade em diferentes distritos sanitários e outra sobre a distribuição espacial e temporal dos casos no município. As variáveis contextuais incluíram: a) índice de Gini; b) proporção de vulneráveis à pobreza, equivalente a pessoas com renda familiar de 1/2 salário mínimo (R$255,00) por residente; c) percentual de pessoas em domicílios sem abastecimento de água e esgotamento sanitário inadequados; d) percentual da população que vivia em domicílios urbanos sem serviço de coleta de lixo; e) percentual da população que vivia em domicílios sem energia elétrica; f) proporção de mulheres chefes de família em vulnerabilidade social; g) índices de desenvolvimento humano (IDH–2010) calculados pela média geométrica dos três subíndices que o compõem e que possuem pesos iguais (Longevidade e esperança de vida ao nascer; renda per capita; Educação/frequência de crianças e jovens da escola - 2/3 – e escolaridade da população adulta - 1/3). Os indicadores foram obtidos no Atlas de Desenvolvimento Humano do Brasil para 2010 (http://atlasbrasil.org.br). Após análise de correlação, os DS foram agrupados em quatro estratos de acordo com a variável explanatória com maior correlação às taxas de mortalidade. As distribuições das frequências e correlações foram obtidas utilizando o software SPSS 20.0.

Para distribuição espacial, os pontos de ocorrência de óbitos foram obtidos nos registros do SIM constantes na DO, utilizando-se os campos “nome” e “endereço”. Posteriormente, os endereços foram localizados no Google Earth Pro e marcados nas unidades de análise ao qual pertenciam. Criou-se um projeto no ambiente de Sistemas de Informações Geográficas (SIG) QGIS 218.15, onde foi configurado o sistema de referência para as coordenadas planas Universal Transverse Mercator (UTM) e Datum SIRGAS2000. A análise de densidade de pontos foi aferida através da técnica de Kernel, com o interpolador utilizando um raio de 100 metros.

A pesquisa foi aprovada pelos Comitês de Ética em Pesquisa da Universidade do Vale dos Sinos (UNISINOS – parecer Nº 3.233.242) e da Secretaria Municipal de Saúde de Porto Alegre (SMS/POA – parecer Nº 3.281.948).

## RESULTADOS

Entre todas as MVHIV no município, 3.164 mulheres foram acompanhadas pelo serviço de monitoramento da transmissão viral do HIV entre 2007 e 2017, sendo identificados 121 óbitos no período. Um caso foi excluído das análises por falta de informações sobre o endereço e a referência em relação à unidade de análise. Maiores percentuais desses óbitos ocorreram entre mulheres com idade entre 30 e 39 anos (43%), de raça/cor preta/parda (55,4%), com um a sete anos de estudo (57%) e que tiveram diagnóstico para HIV em período igual ou inferior a cinco anos antes do óbito (62%). Todos os casos investigados tiveram pelo menos quatro gestações durante a vida.

Entre as causas de óbito, observou-se, em primeiro lugar, as doenças relacionadas ao HIV, representando 77,7% (N = 94) das mortes, e o outros 33,3% (N = 27) distribuídos entre causas variadas, relacionadas às violências (agressão por arma de fogo, objeto cortante ou penetrante ou por força corporal), mortes maternas ou obstétricas, neoplasias, doenças cardiovasculares, ocasionadas por transtornos por uso de substâncias psicoativas, cirrose hepática e artrite piogênica.

Observou-se ainda progressiva elevação do número de óbitos ao longo dos anos, sendo a taxa de mortalidade igual a 0,23/100.000 em 2007 e 4,95/100.000 mulheres em idade fértil em 2017. As maiores proporções de casos ocorreram em MVHIV pretas/pardas, com exceção do ano de 2009 em que a proporção de casos foi maior em mulheres de raça/cor branca (Figura 1).

**Figura 1:** Taxa de mortalidade e proporção de casos por raça/cor em MVHIV, notificadas no SINAN em Porto Alegre/RS entre 2007 e 2017.

Em todo o período, a maior taxa de mortalidade foi registrada no DS Ilhas onde registrou-se 117,83 óbitos por 100.000 mulheres em idade fértil, seguido dos DS Nordeste, Humaitá-Navegantes, Lomba do Pinheiro e Cruzeiro. A menor taxa ocorreu na região Centro Sul com 6,32 óbitos a cada 100.000 mulheres em idade fértil (Tabela 1).

**Tabela 1:** Distribuição taxa de mortalidade em MVHIV notificadas no SINAN, para Porto Alegre/RS e seus Distritos Sanitários, entre 2007 e 2017^a^.

Unidades de Análise	Mulheres idade fértil (10–49)^b^	2007	2008	2009	2010	2011	2012	2013	2014	2015	2016	2017	2007 a 2017
PORTO ALEGRE	444.236	0,23	0,68	2,03	1,58	1,80	2,25	3,15	2,48	3,60	4,50	4,95	27,24
Ilhas	2.546						78,55			39,27			117,83
Nordeste	11.840				8,44		8,44	25,33		8,44	8,44	16,89	76,02
Humaitá/Navegantes	14.690		6,80	6,80	6,80	13,61		6,80		13,61	13,61	6,80	74,89
Lomba do Pinheiro	18.967			5,27	5,27	10,54		5,27	15,81		15,81	15,82	73,81
Cruzeiro	18.858				5,30	5,30	10,60	10,60	5,30	10,60	5,30	5,30	58,33
Leste	35.249			2,84	2,84	2,84	2,84	5,67		8,51	5,67	5,67	36,89
Partenon	35.701		2,80		2,80		5,60	8,40			8,40	5,60	33,61
Eixo Baltazar	30.610					3,27	3,27	3,26	6,53	3,27		9,80	29,40
Glória	18.187		5,50	5,50					5,50	5,50			21,99
Norte	32.648								3,06		12,25	6,12	21,44
Extremo Sul	10.583										9,45	9,45	18,89
Sul	27.781			3,60	3,60				3,60	7,20			17,99
Noroeste	40.124					2,50					4,99	4,99	12,47
Centro	85.050	1,17		3,53				1,17	2,35	2,35		1,17	11,76
Restinga	19.484						5,13				5,13		10,26
Cristal	10.276							9,73					9,73
Centro Sul	31.643			3,16						3,16			6,32

a Por 100.000 mulheres em idade fértil.

b IBGE – 2010.

O coeficiente de correlação bivariada de Spearman indicou correlação moderada entre as taxas de mortalidade de MVHIV com a proporção de mulheres chefes de família por domicílios dos DS e com a proporção de pessoas vulneráveis à pobreza (Tabela 2).

**Tabela 2:** Coeficientes da correlação bivariada de Spearman (p) entre as taxas de mortalidade em MVHIV e variáveis contextuais dos Distritos de Saúde de Porto Alegre.

Indicador/Taxa^a^	Taxa Proporcional de Óbito 2007 a 2017 (p)	IC95%^b^	p
Índice de GINI	0,059	− 0,462–0,586	0,823
% população vulnerável à pobreza	0,558	− 0,031–0,872	0,020
% domicílios sem água e esgoto	0,488	0,009–0,842	0,047
% domicílios sem coleta de lixo	0,543	0,036–0,851	0,024
% domicílios sem energia elétrica	0,281	− 0,253–0,720	0,275
% mulheres chefes de família em vulnerabilidade	0,698	0,215–0,925	0,002
IDHM	− 0,583	− 8,65–0,035	0,014
IDHM – Educação	− 0,562	− 0,878–0,015	0,019
IDHM – Longevidade	− 0,465	− 0,833–0,120	0,060
IDHM – Renda	− 0,502	− 0,808–0,045	0,040

IDHM: Índice de Desenvolvimento Humano Municipal.

a Atlas do Desenvolvimento Humano, 2010.

b Bootstrap por 1000 observações.

O mapa de Kernel (Figura 2) permitiu identificar adensamentos (*hot spots*) nos DS Cruzeiro, Lomba do Pinheiro, Leste e Nordeste. As áreas de menor adensamento foram observadas nos DS Extremo Sul, Centro Sul e Ilhas.

**Figura 2:** Mapa de Kernel dos casos de óbito de MVHIV em Porto Alegre (2007–2017).

A agregação das taxas de óbito, de acordo com o percentual de mulheres chefes de família em vulnerabilidade, demonstrou um gradiente com maiores taxas ocorrendo em distritos onde a vulnerabilidade é maior. As taxas foram 11,92/100.000 para mulheres em situação de menor vulnerabilidade e 83,4/100.000 nas de maior vulnerabilidade (Figura 3). Ou seja, nos DS incluídos no estrato 4 (Nordeste e Ilhas) a mortalidade foi 7 vezes maior em relação ao estrato 1 (Centro, Nordeste, Centro Sul e Sul). Destaca-se que nos DS Nordeste e Ilhas, respectivamente, 37,65% e 34,46% de pessoas residentes viviam em condições de vulnerabilidade à pobreza, possivelmente caracterizando a condição de pauperização dessas mulheres.

**Figura 3:** Distribuição das taxas de mortalidade em MVHIV, de acordo com o percentual de mulheres chefe de família em situações de vulnerabilidade em Porto Alegre (2007–2017).

## DISCUSSÃO

Ainda que avanços tenham sido alcançados com a implementação gratuita da terapia antirretroviral (TARV) no Brasil desde 1996, o quadro de óbitos de MVHIV no município de Porto Alegre revela desigualdades sociais marcantes entre seus DS. As vulnerabilidades contextuais, como pobreza e condições de habitação, somadas à grande presença de mulheres responsáveis pelo sustento familiar e com filhos menores de idade residentes nos DS com taxas elevadas de óbito de MVHIV, retratam as desigualdades sociais. O impacto de tais vulnerabilidades foi identificado por outro estudo em que óbitos por causas relacionadas ao HIV/aids ocorriam mais em pessoas jovens e com piores condições socioeconômicas^12^.

A precocidade do óbitos chama atenção, uma vez que a maior incidência de mortes foi identificada entre as mulheres jovens, com idade entre 30 e 39 anos, corroborando com os dados nacionais para a região sul^6^ e para a população do Rio Grande do Sul^7^. Investigação que avaliou a magnitude e tendência da mortalidade por aids entre 2000 e 2011 (também no Rio Grande do Sul e em Porto Alegre) apontou crescimento dos óbitos de mulheres em idade fértil, de 4,1% (IC95% 3,0–5,3) no estado e de 2,7% (IC95% 1,8–3,5) na capital, sendo que as altas taxas podem refletir a adição de desigualdades sociais às já existentes na distribuição dos serviços assistências^13^.

O munícipio de Porto Alegre apresenta desafios para a gestão da Atenção Primária de Saúde (APS), o que pode se refletir na mortalidade. Nas áreas mais periféricas, onde os índices de violência são elevados, o número de profissionais de saúde é menor em relação às regiões centrais, favorecendo “vazios assistenciais” nas regiões de maior vulnerabilidade^14^. Entre 2006 a 2010, cidades brasileiras com epidemias de Aids de maior magnitude apresentavam contextos de risco relacionados as diferenças de gênero e altas taxas de internação por uso de álcool e drogas^9^, o que desafia as políticas de atenção ao HIV/aids e, particularmente, às mulheres vivendo com HIV.

Diante das características específicas deste estudo, o mapa de Kernel apontou adensamentos em regiões com muitas vulnerabilidades, que apresentam grandes concentrações de pessoas de baixa renda e que recebem até meio salário mínimo^8^, especialmente a região do DS Cruzeiro. Essa região possui grande concentração de sub-habitações, revelando desigualdades na distribuição e consumo dos equipamentos urbanos e na precariedade das condições de existência de seus habitantes^15^. Conforme dados do Censo 2010^8^, as regiões onde se localizam os DS da Lomba do Pinheiro e Nordeste apresentaram IDH abaixo da média nacional, inclusive nos quesitos longevidade, educação e renda.

Os indicadores populacionais mostram que a maior concentração de população negra de Porto Alegre (cerca de 27%^8^) reside em locais com escassez de recursos e saneamento básico^17^, o que impacta negativamente na saúde das mulheres que residem nas regiões Nordeste, Leste, Cruzeiro e Lomba do Pinheiro. Esse fato é importante quando se observa que a maior proporção de óbitos está entres as MVHIV pretas/pardas, revelando a vulnerabilidade dessa população e os efeitos do racismo estrutural no Brasil, resultando em iniquidades relacionadas às mortes^13-16^, geradas pela desigualdade de acesso aos serviços de saúde^17^.

Características como de raça/cor podem influenciar no registro das causas dos óbitos nas DO, podendo causar distorções^18,19^ e, consequentemente, enfraquecer ações direcionadas às populações vulneráveis, devido aos processos de racismo institucional presentes em nossa sociedade^16^. Nos EUA, em 2010, a taxa proporcional de óbitos em negros foi 10 vezes mais alta em relação às pessoas brancas vivendo com HIV^18^. No Brasil, encontrou-se resultado semelhante, onde não brancos eram mais propensos a ter registro de mortalidade por HIV/aids do que indivíduos brancos^20^. Ao mesmo tempo, estudos apontam que o registro de morte para pessoas brancas, cujo diagnóstico HIV foi precoce, tende a não ser relacionada ao HIV/aids, assemelhando-se ao um perfil de causas de óbito da população geral^19^.

As doenças relacionadas ao HIV/aids foram a principal causa de óbito entre os casos deste estudo, corroborando, pesquisa canadense que identificou o HIV/aids como a principal causa de morte entre as pessoas vivendo com HIV e o risco relativo de morte permanece superior ao de indivíduos não infectados^21^. Em que pese os avanços na oferta do diagnóstico e tratamento da Aids, dentre os óbitos de PVHIV de um serviço de saúde carioca, de 1986 a 2009^22^, 73,6% (639/868) foram devido a causas relacionadas à aids, embora esse percentual tenha caído de 86,7% para 61,7% no período. Em Pernambuco, se identificou que 74% (232/315) dos óbitos pesquisados relacionavam-se à imunodeficiência^23^.

Mortes por agressão por arma (de fogo ou cortante) foram a segunda causa de óbito mais prevalente nesta investigação, concorrendo com as causas obstétricas. Os óbitos decorrentes de agressão sublinham os contextos de vulnerabilidade à violência presentes no cotidiano de muitas MVHIV pelo país^24,25^.

Nas regiões das Ilhas e Nordeste as taxas de mulheres com baixa renda e chefes de família são altas e as taxas de óbito entre MVHIV acompanharam essa elevação. Nestas regiões, conforme destaca o Atlas do Desenvolvimento Humano (2010), 1/4 das mulheres residentes são as únicas responsáveis pelo domicílio, não possuem o ensino fundamental completo e têm pelo menos 1 filho com até 15 anos morando no domicílio.

Se por um lado esse resultado reflete a importância crescente das mulheres na subsistência familiar, por outro, indica desigualdades marcantes, já que muitas não tem com quem dividir as responsabilidades da família e arcam sozinhas com o sustento dos filhos^26^. Nessas condições, muitas mulheres podem estar expostas a outras situações de risco na busca por garantir subsistência, envolvendo-se, por exemplo, com tráfico de drogas e outros contextos de violência^26^. Os desafios relacionados ao uso de drogas^27^, podem potencializar barreiras estruturais à vinculação aos serviços de atenção, em especial ao diagnóstico e tratamento precoces do HIV^28^, aumentando o risco de morte entre PVHIV^29^.

Dessa forma, é preciso considerar os desafios que tais vulnerabilidades acarretam quando somadas à baixa capacidade das políticas públicas e dos serviços de saúde em atuar para mitigar tais iniquidades^10,30^. Nesse sentido, estudo^10^ analisando características e itinerários terapêuticos de casos de óbito por Aids, investigados pelo Comitê Municipal de Mortalidade por Aids de Porto Alegre, no ano de 2015, evidenciou-se falhas na assistência e na organização dos serviços de saúde, além de dificuldades de acesso como fatores influentes para os óbitos analisados. Assim, pensa-se em um processo de acumulação, manutenção e até ampliação de desvantagens ao longo da vida que, provavelmente, está presente na maior parte das trajetórias resultantes em óbitos de MVHIV. Ações para redução dos óbitos exigem esforços que considerem as inter-relações entre características psicossociais, culturais e sóciopolíticas^11^, nacionais, locais e regionais para que se minimizem vulnerabilidades e qualifiquem a prevenção de agravos e a assistência em saúde às MVHIV.

Algumas limitações do estudo precisam ser consideradas. A principal limitação dos estudos ecológicos é a impossibilidade de associação entre exposição e desfecho. Os DS como unidades de análise apresentam características heterogêneas; todavia, são definidos pelo SUS e adotados na gestão da saúde em Porto Alegre, sendo que seu uso favorece a tomada de decisão e planejamento de ações nas regiões prioritárias. A dispersão e a heterogeneidade dos dados inviabilizaram a análise de autocorrelação espacial, entretanto, a análise de Kernel permitiu identificar DS de maior adensamento dos casos de óbito. Subnotificações e erros de registro são comuns em bases secundárias e podem afetar a qualidade dos dados, mas o baixo número de informações ausentes nos registros dos óbitos pode indicar a qualificação das fontes de dados utilizados a partir do monitoramento do serviço de vigilância epidemiológica do município.

Adicionalmente, outras causas contribuintes para os óbitos, por sua associação com a infecção por HIV, como a tuberculose, não foram analisadas, pois eventuais problemas de preenchimento dificultariam o cruzamento com outros bancos específicos do SINAN. Ademais, destaca-se que os resultados refletem questões contextuais específicas relacionadas às variáveis utilizadas, não podendo ser extrapolados para o nível individual. Ao assumir as desigualdades sociais, de acesso e de serviços prestados à população de Porto Alegre/RS como aspectos modificáveis, o presente estudo realizou uma análise espacial e espaço temporal de óbitos de MVHIV que foram acompanhadas pelos serviços de atenção à transmissão vertical do HIV no município. Foram analisados aspectos contextuais e epidemiológicos dos DS e as regiões com maior vulnerabilidade social apresentaram maior adensamento dos casos de óbito de MVHIV, revelando o impacto das desigualdades sociais nas taxas de mortalidade dessas mulheres.

Sendo assim, é crucial o enfrentamento e ampliação de esforços no campo das políticas públicas, pela promoção da saúde e prevenção do HIV, sobretudo aquelas que não focalizem somente nas questões reprodutivas^24^, mas que incluam um cuidado integral e longitudinal em saúde das mulheres em geral e, particularmente, das MVHIV. Espera-se que este e outros estudos potencializem ações de atenção e cuidado especialmente direcionados às MVHIV, na provisão de estratégias que ultrapassem intervenções individuais e fomentem políticas públicas comprometidas com a garantia de uma vida digna com acesso à educação, emprego, moradia, renda e cuidados equitativos em saúde.
